# 1-D Compression Behaviour of Acid Sulphate Soils Treated with Alkali-Activated Slag

**DOI:** 10.3390/ma9040289

**Published:** 2016-04-15

**Authors:** Shahidul Islam, Asadul Haque, Ha Hong Bui

**Affiliations:** Department of Civil Engineering, Monash University, Melbourne, VIC 3800, Australia; biplob_orion@yahoo.com (S.I.); ha.bui@monash.edu (H.H.B.)

**Keywords:** 1-D compression behaviour, alkali-activated slag, acid sulphate soil, microstructural analysis, soil improvement

## Abstract

Improvements of soft soils by mechanically mixing cementitious additives have been widely practised for construction of infrastructure. Mixing of additives improves strength and compressibility properties of soils through the development of soil structure. This study investigates the 1-D compression behaviour of alkali-activated slag treated acid sulphate soils (ASS) cured up to 365 days. The void ratio-logarithm of pressure (e-logσ′) behaviour of treated ASS, including the destructuration behaviour, with additive contents and curing time have been analysed. X-ray diffraction (XRD) and scanning electron microscopy (SEM) analyses have been undertaken to explain the observed variations of the 1-D compression behaviour. This paper presents the results of these analyses in view of obtaining an insight into the 1-D compression behaviour of treated ASS with the help of mineralogical analysis.

## 1. Introduction

Improvements of soft soils by mechanically mixing cementitious additives (e.g., lime, cement) have been practised over a long time for the construction of infrastructure. Mixing of cementitious additives imparts structure (bond and fabric) to soils [1], which allows structured soils to exist at higher void ratios than their equivalent reconstituted counterparts [2,3]. When soil stress exceeds the yield stress, it undergoes destructuration through breakage of bonds, and gradually converges to a behaviour similar to its reconstituted counterpart at a relatively high stress when the soil behaviour is controlled by its fabric. In general, the 1-D compression behaviour of soil is represented by the change of void ratio with the logarithm of vertical effective stress (e-logσ′), and is represented by two distinctly different zones: (i) recompression line (pre-yield) whose slope is defined by the recompression index (*C_r_*); and (ii) virgin compression line (post-yield) whose slope is defined by the compression index (*C_c_*). For most practical settlement calculations, these indices are often considered to be constant. However, the compression indices of structured soils vary with vertical effective stresses [2,4,5].

Acid sulphate soils (ASS) are naturally occurring soils, containing sulphide minerals such as pyrite (FeS_2_) up to 15 wt %, and are commonly found along the coastline (approximately 95,000 km^2^) of Australia [6]. ASS are generally under-consolidated materials and construction of infrastructure in ASS are often challenging due to the oxidation of pyrite from the disturbance caused by construction activities. In a recent study, Islam *et al.* [7] reported a reduction of strength for lime-ground granulated blast furnace slag (GGBS) treated ASS resulting from the development of deleterious minerals (thaumasite-ettringite phase). This mineral has been found to be responsible for the degradation of cementitious bonds, which may have an adverse effect on the soil structure and 1-D compression behaviour of treated ASS. The compression behaviour of a treated soil primarily depends on the dose and types of cementitious additives, and the curing periods. However, in the case of ASS, the presence of various quantities of pyrite may also influence the 1-D compression behaviour, which demands a thorough investigation. Therefore, in this study, a series of 1-D compression tests have been carried out on alkali-activated slag (lime-GGBS) treated ASS to investigate the effects of slag, pyrite content and curing time on the compression behaviour of treated ASS. In particular, the changes of yield stress (σ′*_y_*), compression/recompression indices and the process of breaking of bonds with applied stresses, which is known as destructuration, of ASS treated with various proportions of additives and curing time have been discussed. Results of X-ray diffraction (XRD) analysis with quantitative mineral phases and scanning electron microscopy (SEM) including energy dispersive spectroscopy (EDS) have also been incorporated to identify mineralogical changes that are affecting the 1-D compression behaviour of treated ASS.

## 2. Laboratory Investigation

### 2.1. Materials

A natural ASS, locally known as Coode Island Silt, was sampled from the Docklands of Melbourne, Australia. The ASS, which was collected from a depth of 10–12 m, is classified as silt with high plasticity (MH). The mineralogical compositions of ASS including index properties and grain size distributions are shown in Table 1.

Commercially available pyrite powder (85% finer than 60 microns) of 4 wt % was mixed with the natural ASS to simulate responses of higher amounts of pyrite in ASS.

### 2.2. Additives

Hydrated lime and ground granulated blast furnace slag (10 wt % is finer than 2 microns and 99.7 wt % is finer than 63 microns) were used as chemical additives in this study. The chemical compositions (in terms of oxides) of lime and slag are shown in Table 2. The amount of lime required to treat ASS was determined by performing the initial consumption of lime tests [7,8]. A minimum of 12 wt % lime was required for the ASS. Therefore, a slightly higher lime content of 15 wt % for the ASS has been investigated to ensure adequate supply of lime as well as to maintain a favourable reaction environment. For the ASS, a wide range of slag contents (5, 10, 15 and 20 wt %) has been investigated, which included effective slag to lime ratios of 1–1.5 [8,9].

### 2.3. Sample Preparation

ASS was initially mixed with 15 wt % of alkali (hydrated lime) and various proportions of slag (5, 10, 15, 20 wt %). The ASS-additives blend was mixed with 150 wt % of water (≈1.5 × liquid limit) in a mechanical mixer for 30 min to ensure a homogeneous mixture. The prepared mixture was then poured in a cylindrical polyvinyl chloride mould of 50 mm internal diameter and 110 mm height into three layers with a mild vibration around the mould from a hand held vibrator. The bottom and top ends of the mould were sealed with plastic sheets before and after pouring the mixture into the mould, respectively. The prepared samples were then stored in a standard humid chamber for curing up to 365 days. The cured samples were extruded and pushed into a stainless steel cylindrical ring of 50 mm diameter and 20 mm height for 1-D compression tests. The mean initial void ratio of the samples tested in this study was 3.46, with a standard deviation of 0.13. The variation of initial void ratio could have been resulted from the addition of fine pyrite powder and slag with the ASS.

### 2.4. 1-D Compression Tests

Compression tests were carried out on treated ASS samples at different curing periods (30, 90, 180 and 365 days) following AS1289 [10]. In this study, a fully-automated compression rig (LoadTrac-II mounted with a load cell of model SBA-5KLB-I having an accuracy class of III, 5, S/IIIL.10.S) with a maximum stress of 10,000 kPa was used. Tests were conducted under a loading path of 50, 100, 200, 400, 600, 800, 1000, 1200, 1600, 3200, 6400, 8000 kPa followed by an unloading path of 8000, 6400, 3200, 1600, 1200, 800, 400, 200 and 100 kPa.

### 2.5. X-ray Diffraction(XRD) Analysis

XRD analyses were carried out on alkali-activated slag treated ASS at different curing periods (30, 90, 180 and 365 days). Prior to the analysis, the cured samples were dried in an oven at 100 °C for 24 h, and were pulverised and passed through a 425 microns sieve. These samples were then mixed with 10 wt % annealed calcium fluoride (CaF_2_) and subsequently ground under anhydrous ethanol in a McCrone Micronising Mill. XRD data were collected in the School of Chemistry at Monash University using a Bruker D8 Focus θ–2θ X-ray diffractometer (Karlsruhe, Germany). This instrument is equipped with a scintillation detector and a long, fine-focus Cu X-ray tube that was operated at 40 kV and 40 mA. XRD patterns were collected with a step size of 0.02° 2θ and counting time of 2 s/step over the range of 3°–80° 2θ. The constituent mineral phases of treated ASS were identified with reference to the ICDD PDF-2 database and the Crystallography Open Database using the software DIFFRAC^plus^ EVA (Bruker AXS, Karlsruhe, Germany). Quantitative phase analysis was carried out using the Rietveld method [11,12,13]. Rietveld refinements were done with Topas Version 3 [14] using the fundamental parameters approach of Cheary and Coelho [15].

### 2.6. Scanning Electron Microscopy (SEM)

SEM imaging was carried out to study the microstructural development of treated ASS. The SEM and EDS were conducted using a JEOL JSM-7001F FEG SEM equipped with an Oxford Instruments X-Max 80 Silicon Drift type EDS detector (Tokyo, Japan) and Aztec data acquisition software (Oxford Instruments plc, Oxfordshire, UK) at the Monash Centre for Electron Microscopy (MCEM). Crushed specimens of treated samples were imaged using secondary electron imaging (SEI). A platinum coating of 3-nm thickness was applied using a vacuum evaporator prior to the imaging.

A polished thin section of treated sample was prepared in the School of Geosciences of Monash University (Melbourne, Australia) for back-scattered electron imaging (BEI). The thin section was prepared by cutting and polishing the sample into 30-micron sections, each of which was attached to a glass slide with epoxy, and coated with a 3-nm thick layer of carbon. EDS was used for elemental analysis and chemical characterisation of the specimen. Colour model of BEI was also conducted by using EDS detector and Aztec data acquisition software to identify different elements in the treated ASS.

## 3. Results and Discussion

Results of the compression tests for the ASS have been analysed in two stages. Stage 1 deals with the variations of void ratio with logarithm of effective stress (e-logσ′) for different curing time and additive contents. The instantaneous slopes of the e-logσ′ plot, which is defined as the slope between two consecutive pressure points, have been calculated and their variations have been used to explain the destructuration of ASS. Stage 2 deals with the XRD and SEM analyses to explain the observed compression behaviour.

### 3.1. e-logσ′ Behaviour

**Figure 1:**
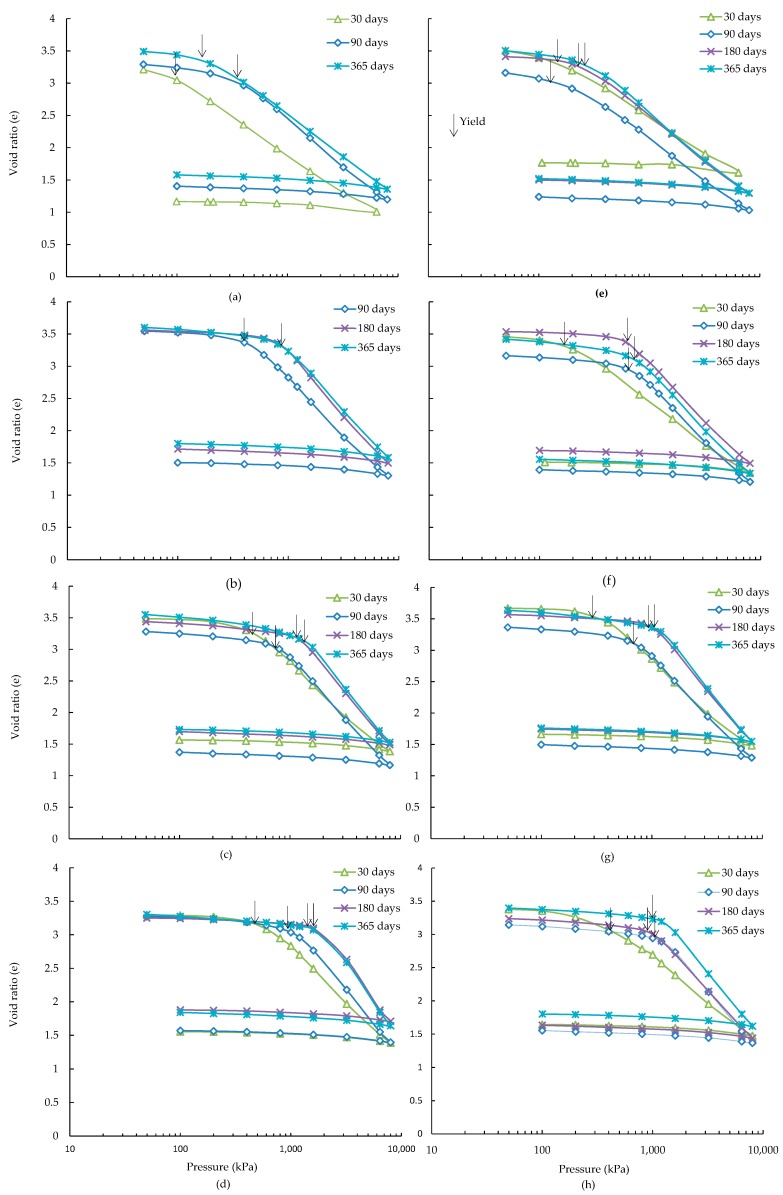
e-logσ′ curves for ASS treated with alkali activated (**a**) 5% slag; (**b**) 10% slag; (**c**) 15% slag; (**d**) 20% slag and for ASS containing 4% additional pyrite and treated with alkali activated (**e**) 5% slag; (**f**) 10% slag; (**g**) 15% slag; (**h**) 20% slag.

Void ratio *vs.* logarithm of effective stress plots (e-logσ′) for all lime-slag treated ASS with curing are shown in Figure 1. It can be seen from this figure that both the recompression and virgin compression behaviour of ASS have been affected by the slag content and curing time due to the formation of various quantities of cementitious reaction products. From the e-logσ′ plots, the slopes of the recompression line (*C_r_*) and virgin compression line (*C_c_*) have been determined (Table 3). In general, the values of *C_r_* were calculated from the slope of the linear part of e-logσ′ plot prior to the yield stress. However, in the case, where inadequate points were found before the yield, the recompression index was calculated from the slope of the recompression line, which may vary slightly from the initial slope of e-logσ′ plot. For the ASS, the *C_c_* has been found to increase with the curing time and slag contents investigated (Figure 1). A similar trend of change of *C_c_* with the curing time and slag content has been encountered for 4 wt % additional pyrite containing ASS treated with up to 15 wt % slag (Figure 1). However, for the 20 wt % slag, the *C_c_* has been found to increase with curing time up to 180 days, and thereafter no change has been observed. In general, the addition of 4 wt % pyrite to ASS decreases the *C_c_* for all curing periods and slag contents investigated (Table 3). On the other hand, the *C_r_* of 5 and 10 wt % slag treated ASS has been found to decrease with the increase of curing time, and no noticeable change has been observed for 15 and 20 wt % slag, particularly at 180 and 365 days. A similar trend of change of *C_r_* with curing and slag have been found for the 4 wt % additional pyrite containing ASS, and treated with up to 15 wt % slag. However, the *C_r_* has been found to marginally increase from 15 to 20 wt % slag for all curing periods. The increase of curing and slag contents is responsible for an increased amount of cementitious reaction products, as discussed in the XRD analysis section, which are believed to affect the development of soil structure as evidenced from an increase of *C_c_* and decrease of *C_r_* values. For the 4 wt % additional pyrite containing ASS, a drop in *C_c_* with an increase of slag contents could be related to the formation of deleterious reaction products (thaumasite-ettringite phase), as identified in the SEM analyses, which is responsible for the degradation of soil structure.

**Figure 2:**
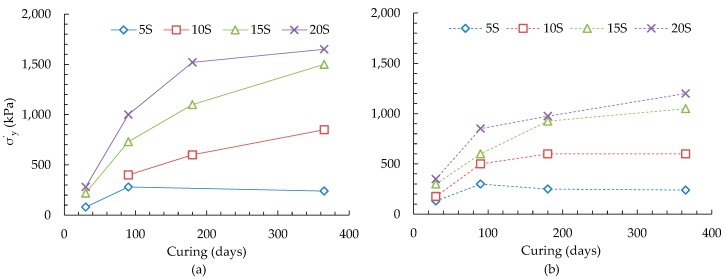
Variations of yield stresses with curing and slag contents for alkali-activated slag treated (**a**) ASS and (**b**) ASS containing 4 wt % additional pyrite.

The yield stress (σ′*_y_*) in this study has been defined as the stress corresponding to the intersection point of two straight parts of the void ratio-logarithm of effective pressure plot. The variations of yield stress of alkali-activated slag treated ASS with curing and additive contents are presented in Figure 2. It can be seen that the yield stress (σ′*_y_*) of ASS treated with 5 wt % slag (slag to lime ratio = 0.33) increases up to 90 days curing and thereafter remains unchanged for up to 365 days (Figure 2a). This may be due to the inadequate supply of slag, which is causing cessation of cementitious reactions after 90 days as evidenced from an insignificant change of amorphous mineral quantities with curing (discussed in Section 3.3). Further increase of slag contents (10 and 15 wt %), with slag to lime ratios of 0.7 and 1.0, shows a considerable increase of σ′*_y_* up to the end of 365 days curing period investigated. This increase of σ′*_y_* may have been associated with the increased amount of cementitious reaction products as observed from an increasing trend of amorphous mineral quantities with curing. Further increase of slag to 20 wt % has been found to increase the σ′*_y_* up to 180 days curing, and thereafter a minimal improvement up to 365 days (Figure 2a) which could be related to the development of thaumasite-ettringite phases as identified in SEM analysis (Section 3.4). On the other hand, a noticeable reduction of σ′*_y_* has been observed for 4 wt % additional pyrite containing ASS treated with all slag contents investigated (Figure 2b). The increase of yield stress for ASS containing 4 wt % additional pyrite has been observed to cease after only 90 days curing when treated with 5 and 10 wt % slag (slag to lime ratios of 0.33 and 0.67), however, for the ASS, the increase was only observed for 5 wt % slag (Figure 2a). Further increase of slag contents (15 and 20 wt %)for ASS containing 4 wt % pyrite, representing slag to lime ratios of 1.0 and 1.33, has been found to increase σ′*_y_* up to the end of 365 days curing but at a relatively slower rate. This shows that a higher slag to lime ratio (≥1.0) is necessary, when lime has been fixed at 15 wt %, to compensate for the negative effect of higher quantities of pyrite in the ASS. The effect of pyrite on the mineralogical development of treated ASS has been investigated and explained with the help of XRD and SEM analyses as discussed in Section 3.3 and Section 3.4.

### 3.2. Destructuration Behaviour

**Figure 3:**
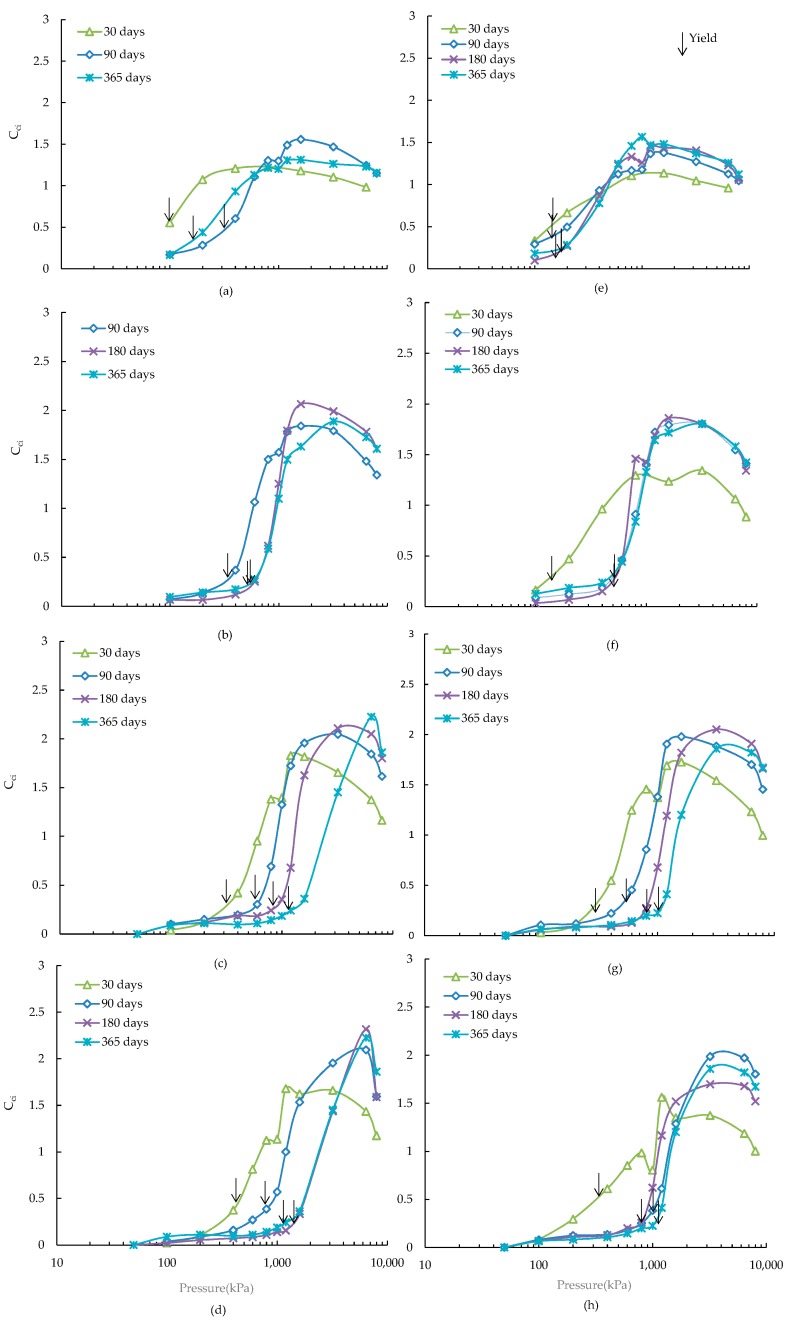
Variations of *C_ci_* with pressure for ASS treated with alkali activated (**a**) 5% slag; (**b**) 10% slag; (**c**) 15% slag; (**d**) 20% slag and for ASS containing 4% additional pyrite treated with alkali activated (**e**) 5% slag; (**f**) 10% slag; (**g**) 15% slag; (**h**) 20% slag.

The variations of instantaneous slope (*C_ci_*) of the e-logσ′ plot with pressure increments can be useful to study the destructuration behaviour of structured clay, a higher value of this parameter indicates a higher amount of destructuration caused by a stress increment [16]. The *C_ci_* values of ASS treated with alkali-activated slag for all the applied stresses were calculated and their variations with effective stresses are shown in Figure 3.

As can be seen, the *C_ci_* *vs.* logarithm of effective pressure up to the yield stress with increased curing lies below those of the shorter curing periods. It can also be seen that the location of the yield points shifts rightward with the increase of curing time. The progressive destructuration occurs immediately after the yield stress with an increase of *C_ci_* values and reaches to a maximum value at a pressure which is defined as the maximum destructuration pressure (σ′*_m_*). The maximum value of *C_ci_* for the ASS has been found to increase with the increase of curing from 30 to 90 days (Figure 3, Table 4), and thereafter a minimal change has been observed up to 365 days curing, which is in good agreement with the results of lime-slag treated ASS reported by Chowdhury [17]. The maximum value of *C_ci_* has been found to increase with the increase of slag from 5 to 10 wt %, and the value has been remained almost unchanged for further increase of slag (Table 4), a similar trend was also observed for cement treated Singapore marine clay [16]. The lower values of maximum *C_ci_* at early stage of curing (30 days) and lower dose of slag (5 wt %) are related to the formation of smaller amount of cementitious minerals, which has been observed in the quantitative mineral phase analyses (Table 5). The presence of additional amount of pyrite has been found to have an insignificant effect (±0.2) on the maximum value of *C_ci_* (Table 4) except for the 20 wt % slag at 180 and 365 days curing, where a drop of maximum *C_ci_* values (up to 0.6) has been observed due to the formation of deleterious minerals (thaumasite-ettringite) as identified in the SEM analyses (Section 3.4).

**Figure 4:**
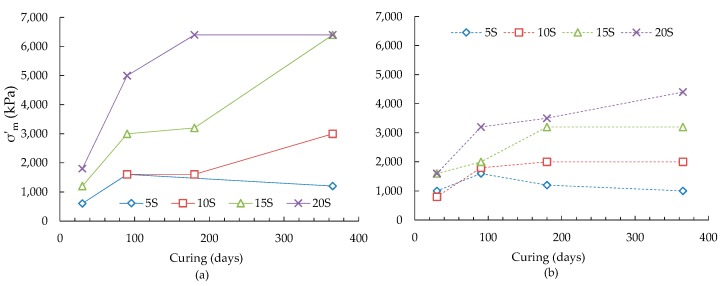
Maximum destructuration stresses with curing and slag contents for alkali-activated slag treated (**a**) ASS and (**b**) ASS containing 4 wt % additional pyrite.

The variations of maximum destructuration stress (σ′*_m_*) with curing and slag contents for ASS are shown in Figure 4.

The maximum destructuration stress has been observed to increase considerably up to 90 days curing for the ASS and thereafter the increase has been dependent on the slag contents. For example, a lower slag content (5 wt %) has contributed to a declined value of destructuration stress (Figure 4a) owing to the cessation of formation of cementitious minerals (Table 5). On the other hand, a higher amount of slag (10, 15 wt %) has contributed to the development of soil structure from the formation of increased amount of cementitious minerals (Table 5) causing an increase of destructuration stress with curing (Figure 4a). Further increase of slag (20 wt %) has resulted in an insignificant change of destructuration stress after 180 days curing due to the oversupply of slag (Table 5). Overall, the addition of 4 wt % pyrite to the ASS has reduced the destructuration stress, particularly at high slag contents (15, 20 wt %). An increase of destructuration stress up to 90 days curing followed by a declined or almost negligible increase afterwards for 5 and 10 wt % slag (Figure 4b) has been observed. Whereas an increase of destructuration stress, with a relatively slower change after 90 days, has been observed for 15 and 20 wt % slag, which could be due to the development of thaumasite-ettringite phase as evidenced from the SEM study.

### 3.3. Mineralogical Quantification

Quantitative phase analysis was carried out by Rietveld refinement of the XRD patterns of ASS treated with various proportions of slag and cured for different time. Disordered phases, such as montmorillonite, and X-ray amorphous nano-crystalline phases, such as cementitious mineral phases (C–S–H), were treated as amorphous phases for the purpose of the Rietveld refinement. The variations of amorphous quantities with curing and additive contents that primarily affect the compression behaviour of treated ASS are shown in Table 5 [8].

Table 5 shows a little variation of amorphous quantities for the ASS treated with 5 wt % alkali-activated slag up to 180 days curing followed by a decreasing trend up to the end of 365 days curing, which appears to have an insignificant impact on the observed yield stresses of treated ASS (Figure 2a). On the other hand, a slow increase in the quantity of amorphous phases up to 180 days of curing for the 5 wt % alkali-activated slag treated ASS containing 4 wt % additional pyrite is observed. This increasing trend is in-line with the slow increase of yield stresses as previously shown in Figure 2b. A relatively constant yield stress associated with almost unchanged amorphous quantities between 180 and 365 days curing (Figure 2b) has been found.

For ASS treated with 10 wt % alkali-activated slag, an overall increase of amorphous quantities from 30 to 365 days of curing is found (Table 5). The increase of amorphous quantity is associated with the increases of yield stress (Figure 2a). However, for the ASS containing 4 wt % additional pyrite, the amorphous quantities are found to decrease slowly (within the observed errors) until the end of 365 days curing. This slow declination of amorphous material quantity may cause to develop relatively smaller yield stresses compared to that of the ASS investigated (Figure 2).

Further increase of slag content (15 wt %) results in an increase of yield stress up to 365 days for both ASS and ASS containing 4 wt % additional pyrite (Figure 2). Although no significant increase in the abundance of amorphous material is observed up to 90 days of curing, the increase thereafter may be due to the formation of poorly crystalline cementitious minerals including thaumasite-ettringite phases resulting from the greater availability of slag, which is affecting the observed changes of yield stresses. These poorly crystalline phases could not be quantified through the Rietveld Refinements using the XRD traces. Therefore, SEM imaging, as discussed below, has been undertaken to further identify these minerals. A similar general trend of change in the refined abundances for amorphous phases and yield stresses is found for 20 wt % alkali-activated slag cured for up to 365 days (Table 5, Figure 2).

### 3.4. Scanning Electron Microscopy (SEM)

**Figure 5:**
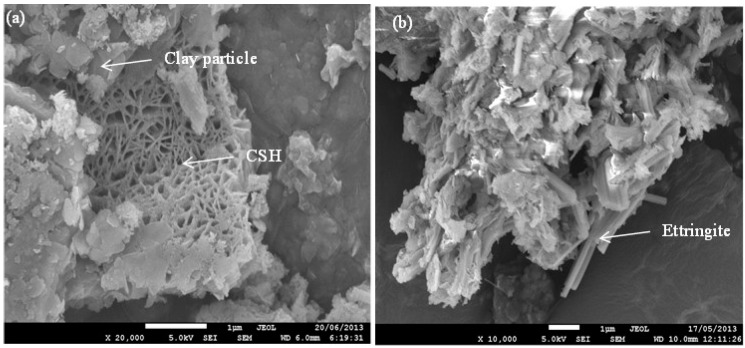
Secondary electron imaging (SEI) of (**a**) ASS treated with 15 wt % lime and 15 wt % slag at 365 days curing and (**b**) ASS containing 4 wt % additional pyrite and treated with 15 wt % lime and 20 wt % slag at 365 days curing.

SEM imaging was undertaken on alkali-activated slag treated ASS specimens containing higher dose of slag (>15 wt %) and cured for longer curing time (365 days), thus allowing the development of cementitious minerals (C–S–H) and deleterious minerals (thaumasite-ettringite). Figure 5a shows the SEI image of ASS treated with 15 wt % slag at 365 days curing. The figure shows that the clay particles transformed from a flaky form into a flocculated structure due to the initial cation exchange process. The rosette-like structures in Figure 5a are cementitious products (C–S–H). The C–S–H and flocculated structure contribute to the increase of yield stress as shown in Figure 2. Figure 4b shows the SEI image of 20 wt % slag treated ASS containing 4 wt % additional pyrite, where the formation of needle-like ettringite has been observed. The ettringite has both strength enhancement and degradation ability [18].

**Figure 6:**
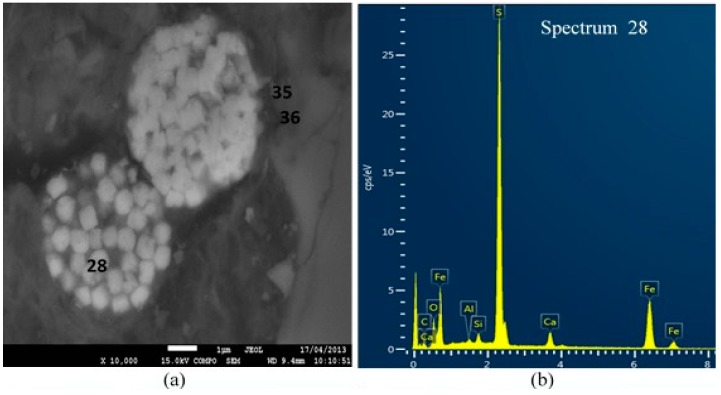

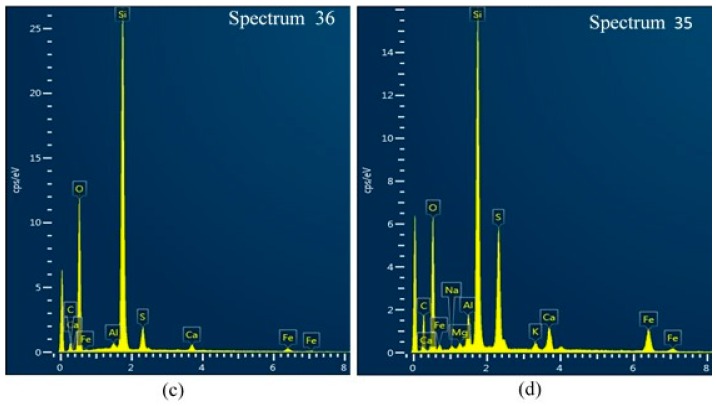
(**a**) Back-scattered electron imaging (BEI) of ASS containing 4 wt % additional pyrite and treated with 15% lime and 20 wt % slag 365 days curing (**b**) energy dispersive spectroscopy (EDS)spectrum at point 28 (pyrite); (**c**) EDS spectrum at point 36 (silica) and (**d**) EDS spectrum at point 35 (thaumasite-ettringite phase).

Figure 6 shows the BEI of 20 wt % slag treated ASS containing 4 wt % additional pyrite. The framboid structure as shown in this figure is consistent with pyrite, which is confirmed by its corresponding EDS spectrum (Figure 6b). On the other hand, the EDS spectrums in the vicinity of the pyrite are consistent with the presence of silica (Figure 6c) and thaumasite-ettringite phases (Figure 6d). The presence of strength-degrading phase, thaumasite-ettringite, can be linked with the smaller yield stress (Figure 2b) for higher pyrite containing ASS.

**Figure 7:**
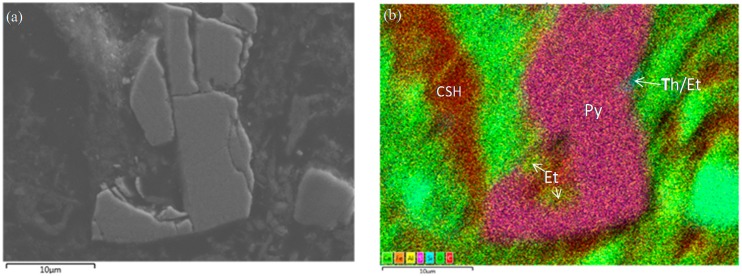
(**a**) BEI and (**b**) BEI colour model of ASS containing 4 wt % additional pyrite and treated with 15 wt % lime and 10 wt % slag at 365 days curing.

Figure 7a shows the BEI of 10 wt % slag treated ASS containing 4 wt % additional pyrite at 365 days curing with its colour model in Figure 7b. In the colour model, the presence of different minerals has been traced through the analysis of combined colour of different elements (Ca, Al, Si, F, S). The colour combination of Figure 7b is consistent with the presence of pyrite (FeS_2_) and C–S–H. In addition, trace quantities of ettringite and thaumasite-ettringite phases are observed in the vicinity of pyrite.

## 4. Conclusions

The 1-D compression behaviour of 15 wt % lime-activated slag treated ASS cured up to 365 days has been investigated in the laboratory with microstructural and mineralogical analyses. The effects of additive content, curing period, and pyrite variations on the compression behaviour of treated ASS were analysed and the findings are summarised below:

- In general, the void ratio-logarithm of effective stress plots of ASS showed an increase of slope of virgin compression line and a reduction of slope of recompression line with increased curing time and slag contents. Similar trend was observed for 4 wt % additional pyrite containing ASS but with an overall reduction of values of these slopes. The yield stress of treated ASS was found to increase with the increase of curing and slag contents, when the slag to lime ratios were 0.67 and 1.0. A higher slag to lime ratios (1.0, 1.33) was required for increasing yield stresses of 4 wt % additional pyrite containing ASS. The increase of yield stresses was associated with the increase of amorphous mineral quantity resulting from the formation of poorly crystalline cementitious reaction products.
- The maximum values of instantaneous slope (*C_ci_*) of the e-logσ′ plots were found to increase with the increase of slag and curing periods. The presence of additional amount of pyrite was found to have a minimal effect on the maximum value of *C_ci_* except for the 20 wt % slag at curing 180 and 365 days, where a drop of maximum *C_ci_* was observed. The drop could be due to the degradation of soil structure resulting from the formation of deleterious minerals (thaumasite-ettringite) as identified through scanning electron microscopy. The stress that causing maximum destructuration was found to increase with the increase of curing time and slag content for the ASS. An overall reduction of maximum destructuration stresses was observed for 15 and 20 wt % slag treated ASS containing 4 wt % additional pyrite.

## Figures and Tables

**Table 1:** Mineralogical constituents, index properties and grain sizes of acid sulphate soils (ASS).

Constituents	wt %	Index Properties	Value
Quartz	24	Liquid Limit	82
K-feldspar	2	Plasticity Index	43
Na/Ca-feldspar	3	pH	7.9
Mica/Illite	10	Cation Exchange Capacity: meq/100 g	33.83
Kaolinite	23	Specific Gravity	2.64
Smectite	32	**Particle Sizes**	
Sulphide	4	Clay (<0.002 mm) (%)	3
Anatase	1	Slit (0.002–0.6 mm) (%)	35
Halite	1	Sand (0.6–2 mm) (%)	62

**Table 2:** Chemical compositions (in terms of oxides) of hydrated lime and lime-ground granulated blast furnace slag (GGBS).

Chemical Compositions (wt %)	Lime	Slag
SiO_2_	1–2	35–37
Al_2_O_3_	0–2	13.5
MgO	–	5.9
Mg(OH)_2_	0.5–1.5	–
CaO	–	41–43
Ca(OH)_2_	85–95	–
Fe_2_O_3_	0–0.7	0.3
SO_3_	–	2.9
MnO	–	0.4

**Table 3:** Compression properties (*C_c_*, *C_r_*) of alkali-activated slag treated ASS.

Slag (%)	5				10				15				20			
Curing (Days)	30	90	180	365	30	90	180	365	30	90	180	365	30	90	180	365
**ASS**																
*C_c_*	1.20	1.50	–	1.71	–	1.85	2.02	2.15	1.76	1.97	2.16	2.22	1.71	2.02	2.50	2.69
*C_r_*	0.14	0.12	–	0.09	–	0.09	0.08	0.07	0.08	0.08	0.07	0.07	0.08	0.07	0.07	0.07
**ASS with 4 wt % Additional Pyrite**																
*C_c_*	1.15	1.35	1.46	1.50	1.32	1.79	1.84	1.87	1.73	1.91	2.14	2.29	1.47	1.98	2.04	2.04
*C_r_*	0.12	0.10	0.09	0.08	0.09	0.08	0.08	0.07	0.08	0.08	0.07	0.06	0.10	0.09	0.07	0.07

**Table 4:** Maximum instantaneous slope (*C_ci_*) of e-logσ′ plot for alkali-activated slag treated ASS.

Slag (%)	5				10				15				20			
Curing (Days)	30	90	180	365	30	90	180	365	30	90	180	365	30	90	180	365
*C_ci_* (max)-ASS	1.2	1.5	–	1.3	–	1.8	2.0	1.9	1.9	2.0	2.1	2.22	1.7	2.1	2.3	2.2
*C_ci_* (max)-ASS + 4% pyrite	1.1	1.4	1.5	1.6	1.3	1.8	1.9	1.8	1.7	1.9	2.0	1.9	1.6	2.1	1.7	1.8

**Table 5:** Change of amorphous quantities (wt %) of treated ASS with curing and slag contents (standard deviations are in brackets).

Slag (%)	Amorphous Quantity (wt %)			
	Curing (Days)			
	30	90	180	365
**ASS**				
5 (σ)	31.9 (3.6)	31.9 (3.6)	30 (3.8)	29 (3.4)
10 (σ)	35.9 (3.4)	39.5 (3.4)	34.8 (3.8)	41.1 (3.2)
15 (σ)	34.2 (4.2)	35.1 (3.2)	35.6 (3.4)	38 (3.4)
20 (σ)	30.9 (3.6)	33.3 (3.4)	34.3 (3.4)	36.1 (3.4)
**ASS + 4% pyrite**				
5 (2σ)	32.5 (3.6)	34.5 (3.4)	35.4 (3.4)	34.4 (3.6)
10 (2σ)	38.7 (3.2)	37.4 (3.4)	34.9 (3.8)	35.8 (3.4)
15 (2σ)	34.1 (3.6)	32.9 (3.4)	34.3 (3.6)	37.1 (3.4)
20 (2σ)	35.9 (3.6)	31.9 (3.6)	33.6 (3.8)	37.9 (3.4)
